# Isoprostanes as Biomarker for White Matter Injury in Extremely Preterm Infants

**DOI:** 10.3389/fped.2020.618622

**Published:** 2021-01-15

**Authors:** Caterina Coviello, Serafina Perrone, Giuseppe Buonocore, Simona Negro, Mariangela Longini, Carlo Dani, Linda S. de Vries, Floris Groenendaal, Daniel C. Vijlbrief, Manon J. N. L. Benders, Maria Luisa Tataranno

**Affiliations:** ^1^Division of Neonatology, Careggi University Hospital of Florence, Florence, Italy; ^2^Department of Medicine and Surgery, University of Parma, Parma, Italy; ^3^Department of Molecular and Developmental Medicine, University of Siena, Siena, Italy; ^4^Department of Neonatology, University Medical Center Utrecht and Utrecht University, Utrecht, Netherlands

**Keywords:** isoprostanes, white matter injury, preterm infants, oxidative stress, brain injury

## Abstract

**Background and Aim:** Preterm white matter is vulnerable to lipid peroxidation-mediated injury. F2-isoprostanes (IPs), are a useful biomarker for lipid peroxidation. Aim was to assess the association between early peri-postnatal IPs, white matter injury (WMI) at term equivalent age (TEA), and neurodevelopmental outcome in preterm infants.

**Methods:** Infants with a gestational age (GA) below 28 weeks who had an MRI at TEA were included. IPs were measured in cord blood (cb) at birth and on plasma (pl) between 24 and 48 h after birth. WMI was assessed using Woodward MRI scoring system. Multiple regression analyses were performed to assess the association between IPs with WMI and then with BSITD-III scores at 24 months corrected age (CA). Receiver operating characteristic (ROC) curve analysis was used to evaluate the predictive value of pl-IPs for the development of WMI.

**Results:** Forty-four patients were included. cb-IPs were not correlated with WMI score at TEA, whereas higher pl-IPs and lower GA predicted higher WMI score (*p* = 0.037 and 0.006, respectively) after controlling for GA, FiO2 at sampling and severity of IVH. The area under the curve was 0.72 (CI 95% = 0.51–0.92). The pl-IPs levels plotted curve indicated that 31.8 pg/ml had the best predictive threshold with a sensitivity of 86% and a specificity of 60%, to discriminate newborns with any WMI from newborns without WMI. IPs were not associated with outcome at 24 months.

**Conclusion:** Early measurement of pl-IPs may help discriminate patients showing abnormal WMI score at TEA, thus representing an early biomarker to identify newborns at risk for brain injury.

## Introduction

Despite improved survival rates of preterm infants in recent years, these infants remain at risk of developing a wide range of complications both in the neonatal period and in the long term. The outcome, furthermore, depends on gestational age (GA) and neonatal morbidity (1, 2). The brain is particularly susceptible to the consequences of preterm birth, which leads to high rates of long-term neurological disabilities. White matter injury (WMI) is the primary type of brain injury in survivors of premature birth (3). Previous magnetic resonance imaging (MRI) studies to determine the spectrum of WMI showed that non-cystic WMI was the most common pattern of brain injury, whereas cystic periventricular leukomalacia (c-PVL) has become uncommon (4, 5).

Furthermore, it has been demonstrated that MRI performed at term equivalent age (TEA) can predict an adverse neurodevelopmental outcome (6). Together with the MRI, the use of specific biomarkers may help in the early diagnosis of WMI to promptly identify those infants who may benefit from early intervention. To date, various studies investigated different biomarkers of brain injury, however the optimal neuro-biomarker able to predict cerebral damage has not been identified (7, 8).

In the pathogenesis of WMI, ischemia and reperfusion, together with inflammation, seem to play a central role in producing reactive oxygen species (ROS) (9). Preterm white matter is highly exposed to oxidative damage because of the imbalance between free radical production and the immaturity of antioxidant enzymes, superoxide dismutases-1 and -2, catalase, and glutathione peroxidase (10–13). The human preterm white matter is especially vulnerable to lipid peroxidation-mediated injury. The aldehydes originated from lipid hydroperoxides can be identified in the glia quantifying F2-isoprostanes (IPs) in human cerebral tissue (14). In addition, IPs are significantly raised in infants with WMI at neuropathological examination (15).

Thus, our study aimed to assess the possible correlation between IPs, measured in cord blood (cb) at birth and plasma (pl) between 24 and 48 h after birth, and WMI evaluated with the MRI at TEA in a cohort of extremely preterm infants. The second aim was to determine the relationship between IPs and neurodevelopmental outcome at 2 years of age.

## Materials and Methods

### Patients

A prospective, observational, single-center study was carried out from September 2012 to September 2014 at the Neonatal Intensive Care Unit of the Wilhelmina Children's Hospital Utrecht, Utrecht, the Netherlands. The study was approved by the local ethics committee (10_365). Preterm infants with a gestational age (GA) below 28 weeks, enrolled after written parental informed consent, and for whom an MRI was successfully performed at TEA, were included in this study. Exclusion criteria were major congenital malformations, chromosomal disorders and inborn errors of metabolism.

### Biomarkers

Blood samples were obtained from the vein of a double-clamped umbilical cord immediately after birth. Heparinized blood samples of 0.5 ml were drawn from the indwelling arterial catheter, inserted for clinical care, during routine tests between 24 and 48 h after birth. All samples were immediately centrifuged in order to obtain platelet-poor plasma and butylated hydroxytoluene (BHT) 1% *w*/*v* in methanol (5 μl per ml of plasma) was supplemented to prevent the *in vitro* lipid peroxidation (16). Samples were frozen at −80°C and stored until shipping to the laboratory of oxidative stress of the University of Siena, Italy, where the analysis was performed. F2-IPs were detected as markers of lipid OS-induced injury according to the LC-MS/MS methodology described by Casetta e al (17).

### MRI Acquisition

The brain MRI was performed on a 3T MR system (Achieva, Philips Medical Systems, Best, Netherlands) at TEA. Infants were wrapped into a vacuum pillow in a SENSE head coil. MiniMuffs (Natus Medical Incorporated, San Carlos, CA, USA) and earmuffs (EM's 4 Kids, Everton Park, Australia) were used for hearing protection. The scanning protocol comprised T2 and T1 weighted imaging. Parameters of the scanning protocol included: axial 3D T1-weighted image (TR = 9.4 ms; TE = 4.6 ms; voxel size 0.94 × 0.94 × 2.0 mm; no gap), coronal 3D T1- weighted image (TR = 9.5ms; TE = 4.6 ms, voxel size 0.78 × 0.91 × 1.2 mm; no gap), axial T2-weighted image (TR = 6,293 ms; TE = 120 ms; voxel size 0.54 × 0.61 × 2.0 mm; no gap), and coronal T2-weighted image (TR = 4,847 ms; TE = 150 ms; voxel size 0.78 × 0.89 × 1.2 mm; no gap). Respiratory rate, heart rate and arterial oxygen saturation were monitored during MRI, and a neonatologist or physician assistant was present during the examination. When necessary, infants were sedated using oral chloral hydrate using a dose of 50 mg/kg through the gastric tube. The severity of brain injury was assessed by using conventional coronal T1- and T2-weighted spin-echo sequences. All MRI images were scored for the presence of brain abnormalities by two experienced neonatologists (LdV and MB) with more than 20 years' experience in neonatal neuroimaging, using the Woodward scoring system (6). This scoring system evaluates the presence/severity of white matter volume loss, white matter signal abnormality, the presence of cystic lesions, ventricular dilation, thinning of the corpus callosum and reduced myelination. The white matter score classes were then calculated based on the infant's total scores: normal, mildly abnormal, moderately and severely abnormal.

### Clinical Data Collection

Clinical and demographic parameters were collected from patients' medical records: GA, birth weight (BW), BW <10th percentile, gender, mode of delivery, Apgar score at 5 min of life, fraction of inspired oxygen (FiO_2_) between 24 and 48 h after birth, occurrence and duration of non-invasive respiratory support and mechanical ventilation, sepsis, bronchopulmonary dysplasia (BPD), necrotizing enterocolitis (NEC), intraventricular hemorrhage (IVH), retinopathy of prematurity, and length of stay in the hospital. Sepsis was defined as positive blood culture. BPD was defined as oxygen requirement at 36 weeks of PMA (18). NEC was defined as Bell's stage >2 (19). IVH was classified according to Papile et al. (20). Socioeconomic status was determined based on maternal educational level (21).

### Neurodevelopment Assessment

Neurodevelopmental outcome was assessed at 2 years corrected age (CA) using the Bayley Scales of Infant and Toddler Development, 3rd edition (BSITD-III). Cognitive and motor composite scores were used (mean in a normative population 100, standard deviation 15).

### Statistical Analysis

Patients' characteristics were described as mean and standard deviation (SD), rate and percentage, or median and interquartile range (IQR). Changes in IPs levels were compared using Wilcoxon signed rank test. Univariate regression analyses were performed to assess the association between cb-IPs and pl-IPs with WMI. For the cb-IPs analysis the following variables were entered into the model: GA, BW (percentile) and severity of IVH. For the pl-IPs analysis mean FiO_2_ at sampling was entered into the model instead of BW (percentile). GA and birth weight were included into the regression model because an inverse correlation with IPs has been documented (22, 23). FiO_2_ was entered since previous studies reported an association between the use of higher oxygen concentrations and increased levels of oxidative stress biomarkers (24, 25). Then, IVH was considered because pl-IPs have been found associated with higher degree of IVH (26). A *p* < 0.05 was considered statistically significant. Results were presented as coefficients of the independent variables with the 95% confidence intervals (CI). Lastly, multivariate regression models were performed to examine the association between cb-IPs and pl-IPs with BSITD-III scores at 24 months CA. In these analyses, data was corrected for GA, WMI and socio-economic status. Since in our population only one infant showed severe IVH, we included WMI instead of IVH in the analysis. Condition indexes and the variance inflation factor (VIF) of the regression model were computed to detect multi-collinearity. Condition Index <30 and VIF variance inflation factor <5 values indicated that regression models did not have significant multicollinearity. Data analysis was performed using IBM SPSS Statistics version 20 (SPSS INC, Chicago, Illinois, USA). To analyse the impact of the pl-IPs level on the occurrence of any WMI, ROC (receiver operating characteristic) curve analysis was used. The test's ability to classify patients as those who will develop WMI or not is represented by the area under the ROC curve (AUC).

## Results

Clinical characteristics of enrolled infants (*n* = 44) are summarized in Table 1. cb-IPs and pl-IPs levels were not significantly different [54.2 (36.4–79.2) vs. 58.6 (38.2–83.2)] (*p* = 0.424). Twenty-seven (61%) of patients had mild WMI, and eight (18%) developed moderate WMI. No infants had severe white matter abnormalities on MRI. Forty-two infants returned for follow up examination. The mean scores for cognitive (101 ± 12) and motor (106 ± 13) outcomes were well within the normal range.

**Table 1:** Clinical characteristics of studied infants.

	***N* = 44**
Gestational age (weeks), mean (SD)	26.4 (1)
Birth weight (g), mean (SD)	889 (182)
Birth weight <10th percentile^°^, *n* (%)	4 (9)
Male, *n* (%)	23 (52)
Cesarean section, *n* (%)	23 (52)
Apgar score 5 min, median (IQR)	8 (7–8)
Mechanical ventilation (days), median (IQR)	6 (2–19)
BPD, *n* (%)	11 (25)
PDA, *n* (%)	
Pharmacological treated	22 (50)
Surgical closure	3 (7)
Sepsis, *n* (%)	5 (11)
NEC, *n* (%)	
Conservatively treated	1 (2)
Surgery	3 (7)
IVH, *n* (%)	
1-2 grade	15 (34)
3-4 grade	1 (2)
Cerebellar hemorrhages, *n* (%)	
Large unilateral/bilateral lesion or vermis involvement	0 (0)
Punctate hemorrhages	7 (16)
PMA at scan (weeks), mean (SD)	40.9 (1)
Posthemorrhagic ventricular dilatation requiring intervention, *n* (%)	1 (2)
WMI score, *n* (%)	
No	9 (21)
Mild	27 (61)
Moderate	8 (18)
Cognitive score at 24 months^*^, mean (SD)	101 (12)
Motor score at 24 months^*^, mean (SD)	106 (13)
Maternal education (low/middle/high), n (%)	10/20/12 (24/48/28)
FiO_2_ between 24 and 48 h after birth, mean (SD)	25.5 (5.0)
Plasma isoprostanes (pg/ml), median (IQR)	54.2 (36.4–79.2)
Cord blood isoprostanes (pg/ml), median (IQR)	58.6 (38.2–83.2)

° Birth weight percentile was computed according to the Dutch Perinatal registry reference data (27); BPD, bronchopulmonary dysplasia; PDA, patent ductus arteriosus; Sepsis, culture proved sepsis; NEC, necrotizing enterocolitis; IVH, intraventricular hemorrhage; PMA, postmenstrual age; WMI, white matter injury, evaluated according to the scoring system by Woodward et al. (6)

* *Bayley Scales of Infant Development, third edition*.

*Mean (SD), number (percentage) or median (IQR)*.

### Cord Blood Isoprostanes

Univariate regression analysis demonstrated that cb-IPs were not associated with WMI at TEA. After controlling for GA, BW (percentile) and severity of IVH, lower BW was associated with a higher WMI score (*r* = 0.65; *p* = 0.025) (Table 2). cb-IPs were not associated with cognitive and motor outcome at 24 months CA. After controlling for GA, maternal education and WMI, maternal educational level was associated with higher cognitive scores (*r* = 0.63; *p* = 0.001) (Table 3).

**Table 2:** Multivariable linear regression analysis between cb-IPs and WMI score at TEA.

	**WMI**		
	***R*** **=** **0.65**		
	**B**	**95% CI**	***p***
**Gestational age**	0.05	−0.22−0.34	0.682
**BW (percentile)**	−0.011	−0.020−−0.001	**0.025**
**Any IVH**	0.2	−0.1−−0.4	0.243
**cb-IPs**	−0.002	−0.006−0.001	0.158

*Bold values are those statistically significant (p < 0.05)*.

**Table 3:** Multivariable linear regression analysis between cb-IPs and neurological outcome at 24 months CA.

	Cognitive score 24 months^*^			Motor score 24 months^*^		
	***R*** **=** **0.63**			***R*** **=** **0.30**		
	**B**	**95% CI**	***p***	**B**	**95% CI**	***p***
Gestational age	2.7	−4.2–9.8	0.418	−2.6	−10.3–5.1	0.556
Maternal education	8.2	3.7–13.2	**0.001**	1.4	−4.7–7.5	0.631
WMI	0.2	−3.8–4.4	0.861	2.7	−1.8–7.4	0.224
cb-IPs	0.004	−0.077–0.085	0.926	0.045	−0.042–0.102	0.393

* *Bayley Scales of Infant Development, third edition*.

*Bold values are those statistically significant (p < 0.05)*.

### Plasma Isoprostanes Between 24 and 48 h After Birth

**Figure 1:**
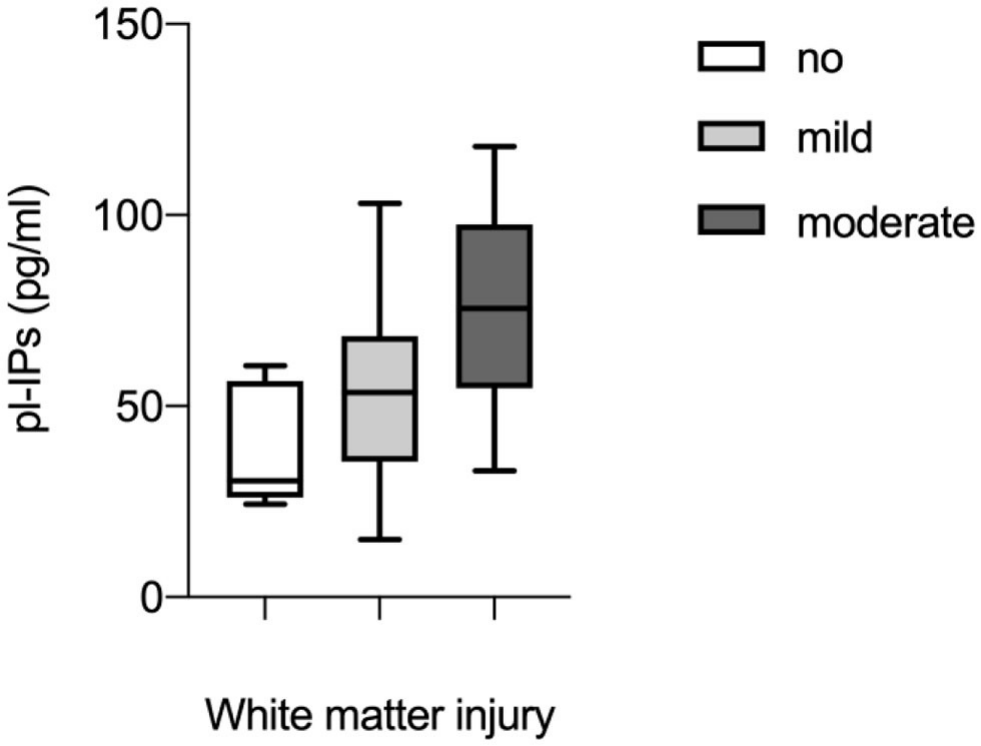
Correlation between plasma levels of isoprostanes (pl-IPs) between 24 and 48 h after birth and WMI evaluated according to the scoring system by Woodward et al. (6). Multivariable regression analysis (*p* = 0.037).

Univariate regression analysis demonstrated that pl-IPs were positively associated with WMI at TEA (*r* = 0.39; *p* = 0.02). After adjusting for GA, FiO2 at sampling and severity of IVH, higher pl-IPs concentration and lower GA were associated with a higher WMI score (*r* = 0.65; *p* = 0.037 and 0.006, respectively) (Table 4; Figure 1).

**Table 4:** Multivariable linear regression analysis correlating pl-IPs between 24 and 48 h after birth and WMI score at TEA.

	**WMI**		
	***R*** **=** **0.65**		
	**B**	**95% CI**	***p***
Gestational age	−0.5	−1.0−−0.5	**0.006**
FiO_2_	0.009	−0.084−0.103	0.843
Any IVH	0.2	−0.4−−0.9	0.523
pl-IPs	0.015	0.001−0.032	**0.037**

*Bold values are those statistically significant (p < 0.05)*.

**Figure 2:**
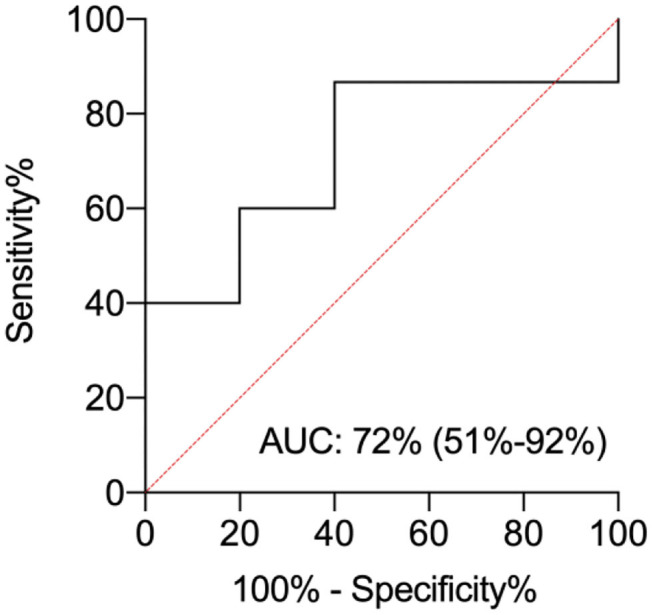
ROC curve analysis for plasma isoprostanes (pl-IPs) concentration between 24 and 48 h after birth. The area under the curve is 0.72 (C.I. 95%= 0.51–0.92). The pl-IPs levels plotted curve indicated 31.8 pg/ml as the best predictive threshold with a sensitivity of 86% and a specificity of 60%. ROC curve discriminates newborns with any WMI from newborns without WMI.

The area under the curve (AUC) was 0.72 (C.I. 95% = 0.51–0.92). The pl-IPs levels plotted curve indicated that 31.8 pg/ml had the best predictive threshold with a sensitivity of 86% and a lower specificity of 60% to discriminate newborns with and without WMI (Figure 2).

pl-IPs were not associated with cognitive and motor outcome at 24 months CA. After controlling for GA, maternal education and WMI, maternal education level was associated with higher cognitive scores (*r* = 0.52; *p* = 0.004) (Table 5).

**Table 5:** Multivariable linear regression analysis correlating pl-IPs between 24 and 48 h after birth and neurological outcome at 24 months CA.

	Cognitive score 24 months^*^			Motor score 24 months^*^		
	***R*** **=** **0.52**			***R*** **=** **0.27**		
	**B**	**95% CI**	***p***	**B**	**95% CI**	***p***
Gestational age	2.6	−1.9–7.3	0.243	−3.0	−9.2−3.2	0.328
Maternal education	6.0	2.1–9.9	**0.004**	0.08	−5.8–6.0	0.977
WMI	−1.0	−4.9–2.4	0.544	2.0	−2.9–6.9	0.402
pl-IPs	0.041	−0.10–0.18	0.573	−0.14	−0.34–0.05	0.135

* *Bayley Scales of Infant Development, third edition*.

*Bold values are those statistically significant (p < 0.05)*.

## Discussion

Our study investigated the association between IPs levels in the early postnatal period in extremely preterm infants and WMI evaluated at TEA with MRI and neurological outcome at 24 months CA. In this cohort, 79% of the neonates had mild to moderate WMI visible on their MRI at TEA. Our results revealed that early pl-IPs concentration was higher in infants with WMI, and this correlation remained statistically significant after adjusting for potential confounding factors. Thus, pl-IPs levels might be a valuable early biomarker of later WMI.

In preterm infants, during the developmental window between 23 and 32 weeks PMA, the brain is at significant risk for WMI because the white matter is exposed to oxidative stress due to hypoxia-ischemia or maternal-fetal infection (28, 29). Pre-oligodendrocytes (OLs) dominate the white matter during this phase, with the first appearance of differentiated OLs around 30 to 35 gestational weeks (30). Studies of cultured oligodendroglial cells indicate that pre-OLs appear particularly prone to free radical-mediated injury because of the immaturity of antioxidant defenses, whereas differentiated OLs are more resistant to oxidative stress both *in vitro* and *in vivo* (10–12, 15, 31, 32). ROS released by activated microglia interact with cell structures like lipids and membranes, inducing lipid peroxidation that results in the release of highly reactive derivatives (33). IPs are the result of free radical-induced injury by peroxidation of lipids in cell membranes. They are formed *in vivo* by the peroxidation of arachidonic acid, mediated by free radical production by a non-cyclooxygenase mechanism (34, 35). IPs are chemically stable compounds and have been detected in fluids and tissues, such as in the brain, where IPs are enriched in glial cells and are sensitive and specific biomarkers of oxidative stress in adult human brain tissue from autopsy (36, 37). Furthermore, IPs are bioactive compounds that may exert vasoconstriction of different vascular beds, including the brain (38). Thus, it has been hypothesized that IPs may participate in neurovascular injury. To support this theory, IPs have been shown to induce neuromicrovascular endothelial cell death via thromboxane (TXA2) production in rat and piglet models of hypoxia-ischemia encephalopathy (39). Brault et al. demonstrated that IPs provokes pre-OLs death by oncosis, depending on increased production of TxA2 and inadequate antioxidant defenses, whereas little effect has been demonstrated on mature OLs (40).

Back et al. quantified oxidative damage directly in human preterm autopsy brains and found that the increase of IPs concentration generated during the early phases of WMI was associated with a marked depletion of the pre-OL. Moreover, the IPs levels were comparable to those found in the cerebral cortex after severe perinatal asphyxia in term infants (15). A further finding was that IPs concentration was not elevated in cerebral cortex, this is consistent with the concept that WMI develops from hypoxia–ischemia related to vascular end-zones. Hence, oxidative injury selectively targeted the white matter and spared the cerebral cortex during ischemic events. In a previous study, an increase in IPs concentration was demonstrated in the cerebrospinal fluid of 14 infants with WMI evaluated on MRI at TEA, with a strong increase in those with more severe cystic PVL with ventriculomegaly compared to infants with less severe forms of WMI such as white matter signal abnormality or ventriculomegaly alone (41). In addition, pl-IPs were significantly augmented during the first week after birth in preterm infants who went on to develop c-PVL and severe IVH (26).

We found that pl-IPs cutoff level of 31.8 pg/ml measured between 24 and 48 h after birth showed a good sensitivity for WMI (86%) but a low specificity (60%). The small number of subjects enrolled might have influenced this finding. Furthermore, previous studies correlated elevated levels of lipid peroxidation products and several complications of prematurity besides WMI. In fact, early pl-IPs concentration was found higher in infants who later developed BPD or died and severe IVH compared to survivors without (26). In addition, high urinary excretion of IPs has been associated with hemodynamically significant PDA in preterms <34 weeks GA (42). BPD, IVH, PDA, and WMI, all belong to the “free radicals disease” of prematurity, disorders caused by the harmful effect of free radicals (43, 44).

According to our results, pl-IPs predicted WMI at TEA, but not IPs measured on cord blood. cb-IPs reflect oxygen radical exposure during fetal life and placental metabolism of lipid peroxides. It has been reported that IPs concentration varied according to prenatal and precocious infants factors such as chorioamnionitis, fetal inflammatory response of the placenta, perinatal depression, fetal growth and birth weight (23, 45). On the other hand, early pl-IPs concentration might be influenced by other perinatal and postnatal factors, such as FiO_2_ during resuscitation in the delivery room, respiratory distress syndrome and mechanical ventilation, leading to an increased production of oxygen free radicals (24, 46–48).

In our model, lower BW was correlated to higher WMI score, probably due to lower GA. This result might be related to intrauterine growth restriction since this condition was diagnosed to the four infants small for gestational age (BW percentile <10th) enrolled in our study. Previous findings reported that fetal growth restriction has been associated with delayed oligodendrocyte maturation and myelination in animal models, and diffuse white matter abnormality in preterm infants (49, 50).

We did not find an association between IPs concentration in cord blood and plasma with neurological outcome. On the contrary, Matthews et al. demonstrated increased pl-IPs in preterm infants at risk for worse developmental outcomes adjusting for GA, maternal education and severe abnormalities on neuroimaging (51). The possible explanation for these conflicting results might be the higher incidence of severe abnormalities on neuroimaging [c-PVL, severe IVH (grade III or IV) and cerebellar hemorrhage], compared to our population. Furthermore, the different timing of sampling could also play a role as Matthews analyzed the change in pl-IPs concentration between 14 and 28 days after birth.

Additionally, we failed to find a correlation between WMI at TEA and BSITD-III at 2 years CA. Several studies have proven that MRI executed at term equivalent age (TEA) can predict an adverse neurodevelopmental outcome (6, 52, 53). The lack of this result might reflect the characteristic of our cohort, since none of the newborns showed severe brain injury and at follow up examination the scores were in a regular range. We found that maternal educational level was significantly correlated with cognitive development. This finding suggests that both genetic and environmental factors influence neurodevelopmental outcome. However, other covariates that were not analyzed due to the small sample size might also have influenced early development.

This study has some limitations. First, with the determination of IPs, we explore a specific type of brain injury, involving the prostaglandin metabolism. Other products of lipid peroxidation such as neuroprostanes, IPs-like molecules from docosahexanoic acid (DHA) might be an attractive and more specific biomarker of brain injury since DHA is more abundant than arachidonic acid in the brain (54, 55). The second limitation is the small sample size, which may have limited the possibility to detect a significant role of other potential mediating or interactive factors on outcome. Additional studies with a *larger* sample size, including infants with more severe brain injury, are needed in order to better define the most predictive cut-off value of pl-IPs for WMI detection.

## Conclusion

Early measurement of pl-IPs may help to identify patients with a higher chance of an abnormal WMI score at TEA. The pl-IPs levels plotted curve indicated that 31.8 pg/ml had the best predictive threshold with a sensitivity of 86% and a lower specificity of 60%. pl-IPs might represent a reliable biomarker useful to identify newborns at high risk for brain injury. However, further researches on larger population of infants are needed to validate these findings.

## Data Availability Statement

The raw data supporting the conclusions of this article will be made available by the authors, without undue reservation.

## Ethics Statement

The studies involving human participants were reviewed and approved by Pediatric ethics committee of the Wilhelmina Children's Hospital Utrecht, Utrecht, Netherlands. Written informed consent to participate in this study was provided by the participants' legal guardian/next of kin.

## Author Contributions

CC, SP, GB, SN, ML, CD, LV, FG, DV, MB, and MT have made substantial contributions to conception, design of the study, critically revised the article, and have given their final approval of the version to be published. CC and MT performed the data statistical analyses and wrote the manuscript. CC, MT, and SN collected data. All authors contributed to the article and approved the submitted version.

## Conflict of Interest

The authors declare that the research was conducted in the absence of any commercial or financial relationships that could be construed as a potential conflict of interest.
